# Properties of Sunflower Straw Biochar Activated Using Potassium Hydroxide

**DOI:** 10.3390/molecules30061332

**Published:** 2025-03-16

**Authors:** Siyu Chang, Lei Wang, Lihong Yao

**Affiliations:** 1 College of Materials Science and Art Design, Inner Mongolia Agricultural University, Hohhot 010018, China; csy792111@emails.imau.edu.cn; 2 Inner Mongolia Autonomous Region Russian and Mongolian Imported Wood Processing and Utilization Engineering Technology Research Center, Hohhot 010018, China

**Keywords:** biochar, potassium hydroxide, activation, sunflower straw

## Abstract

Biochar is a kind of carbon material with a wide range of sources; it has attracted considerable attention because of its abundant resources and low cost. Potassium hydroxide (KOH) is a strong alkali activator that can effectively change the surface chemical properties and microstructure of biochar. Biochar activated by KOH has a large specific surface area (SSA) and a rich pore structure. Herein, sunflower straw was used as a raw material and KOH as an activator to investigate the preparation of sunflower straw biochar activated by KOH. The effects of synthetic conditions on the performance and structure of the resulting biochar materials were comprehensively analyzed. The final activation conditions were as follows: the impregnation ratio, activation time, and activation temperature were 2:1, 2 h, and 900 °C, respectively. The composition and structure of the prepared biochar were characterized. It was observed by SEM that the surface of the activated biochar became rougher. FTIR, XRD, XPS, and Raman characterization showed that the aromaticity and graphitization degree of the activated biochar increased. The activation process of biochar was analyzed via multiple techniques, aiming to lay the foundation for the wide application of biochar materials.

## 1. Introduction

Sunflower is an annual herbaceous plant of the Asteraceae family, which is one of the most important and valuable oil crops. Sunflower seeds have a high oil content, and sunflower seed oil is the main edible oil in many countries [1]. With increasing demand for sunflower seed oil, the number of sunflowers planted has been steadily increasing every year [2]. During the processing and utilization of sunflowers, large amounts of by-products are generated—around 120 million tons of sunflower straw annually [3]. The utilization rate of sunflower straw is low, and it is usually incinerated. Thus, a large number of resources are not fully utilized, causing environmental pollution. For this reason, the high-value-added utilization of sunflower straw can not only save natural resources but also practically contribute to environmental protection and promote the efficient development of the sunflower industry [4,5,6].

The preparation of biomass-derived carbon materials is one of the important methods for the value-added utilization of biomass raw materials. Biomass-derived carbon is a carbon material with a large SSA and rich pores. Wang et al. used KOH activation combined with pyrolysis to prepare peanut shell biochar material. The study observed that the pore structure and SSA of biochar were significantly improved. The prepared biochar had a mesoporous–microporous structure and an SSA of 158.69 m^2^/g [7]. Feng et al. prepared corncob biochar by using phosphoric acid, biological oil, and wood vinegar as activators. The results showed that the specific surface area and pore volume of the corncob biochar prepared with phosphoric acid as activator were the largest. When the activation temperature was 850 °C, the pore volume and SSA were 0.3 cm^3^/g and 657.76 m^2^/g, respectively. In addition, it was found that the content of carboxyl groups gradually decreased with an increase in activation temperature [8]. Suman et al. analyzed the effect of temperature (1000, 800, 600, 400 °C) on the properties of coconut biochar. They found that pyrolysis temperature affected the functional groups, pH, SSA, and carbon content of coconut-based biochar materials. Interestingly, the yield of coconut biochar, the content of H, and the ratio of H/C decreased with increasing pyrolysis temperatures. However, the SSA, ash content, carbonization degree, and pH value increased [9]. Cao et al. prepared bagasse biochar by combining microwave pyrolysis with KOH activation. It was found that the yield of bagasse biochar decreased from 31.41% to 11.8% with increases in KOH content and pyrolysis temperature, while the SSA initially increased and subsequently decreased [10].

Potassium hydroxide (KOH) is a very common chemical activation reagent, often used to increase the SSA of biochar. During the activation of biochar, numerous micropores are formed and tar production is suppressed, effectively preventing pore blockage [11,12]. Some studies have focused on the activation effect of KOH on biochar. For example, Andas et al. treated palm kernel shells with KOH and obtained biochar with a rich pore structure. Their optimal modification conditions were as follows: an activation temperature of 800 °C and an impregnation ratio (raw biomass–KOH) of 1:1.5 [13]. Feng et al. activated sugarcane bagasse biomass with KOH, yielding a biochar material with a large pore volume (1.34 cm^3^/g), high SSA (2296 m^2^/g), and multilevel porosity [14]. Doczekalska found that biochar treated with KOH contained abundant chemical groups [15]. Tian et al. prepared high-performance porous carbon through a low-temperature alkaline melting–KOH activation method, with the resulting material exhibiting an SSA of 2167.5 m^2^/g. The above findings indicate that the treatment of biomass carbon with KOH increases its SSA and improves its performance in various applications [16].

Herein, we investigated the structure and properties of KOH-activated sunflower straw biochar (KBC) prepared under different activation conditions. Our analysis mainly focused on the effects of impregnation ratio (KOH–sunflower straw biochar (SFBC) ratio), activation time, and temperature on the properties of KBC, including the SSA, yield, water absorption, and elemental composition. Various instruments were used to characterize the structure and composition of the KBC. The activation conditions were determined by comparing the SSA, yield, water absorption, and elemental composition of KBC activated under different conditions. This study aims to provide basic theoretical guidance for promoting the high-value-added utilization of biomass resources and lays the foundation for its application in the field of energy and environmental field.

## 2. Results and Discussion

### 2.1. Effects of the Impregnation Ratio

**Figure 1:**
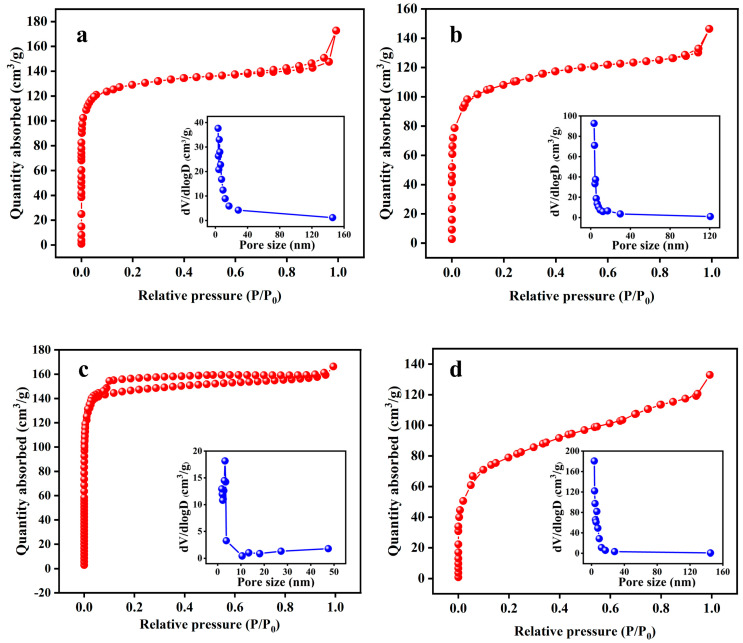
BET and BHJ pore-size distributions of KBC materials with various impregnation ratios: (**a**) 0.5:1, (**b**) 1:1, (**c**) 2:1, and (**d**) 3:1.

First, the effects of various impregnation ratios (0.5:1, 1:1, 2:1, and 3:1), with the activation time and temperature fixed at 700 °C and 2 h, on the properties (SSA, yield, water absorption, and elemental content) of KBC were obtained. The N_2_ adsorption–desorption isotherms of KBC_0.5:1_, KBC_1:1_, KBC_2:1_, and KBC_3:1_ (Figure 1) were all type-I isotherms. When *P*/*P*_0_ was below 0.1, the adsorption capacity of KBC rapidly increased with an increase in *P*/*P*_0_, indicating that the KBC contained numerous micropores. When *P*/*P*_0_ was between 0.1 and 0.9, the adsorption capacity of the KBC slowly increased with increasing *P*/*P*_0_ and the isotherms gradually become horizontal. This indicated that the KBC contained a few mesopores or macropores. When *P*/*P*_0_ approached 1, the adsorption amounts slightly increased owing to capillary condensation in large pores [17]. According to the BHJ pore-size distribution, the pores in KBC_0.5:1_, KBC_1:1_, KBC_2:1_, and KBC_3:1_ mainly had sizes between 3.18 and 3.40 nm.

Notably, the SSA of the KBC was considerably higher than that of SFBC. KBC_2:1_ showed the highest specific surface area, reaching 594.655 m^2^/g (Table 1). This indicated that KOH, as an activator, separated the three major components (cellulose, hemicellulose, and lignin) of sunflower straw and promoted the formation of a microporous structure [18,19]. Activation using KOH included two processes: thermal decomposition on the material’s surface and etching due to KOH. When small amounts of KOH were added, pyrolysis and etching occurred simultaneously. Meanwhile, when large amounts of KOH were added, etching was the predominant process [20]. At an impregnation ratio of 0.5:1, the SFBC was not fully activated because of insufficient KOH addition. Under these conditions, the SFBC was primarily activated by pyrolysis, leading to a weak pore-formation effect. At an impregnation ratio of 2:1, the SFBC was well activated, with etching being the dominant process, resulting in the production of large amounts of CO, H_2_, H_2_O, and other substances, as well as the formation of pores on the SFBC [21]. However, at an impregnation ratio of 3:1, K_2_O and the excess KOH generated by the activation reaction reacted with the skeletal C atoms around the micropores, causing pore collapse and reducing the specific surface area of the KBC [22]. In this case, thermal decomposition and etching occurred simultaneously, competing with each other and reducing the SSA of the KBC [23].

With an increase in the impregnation ratio from 0.5:1 to 3:1, the yield of KBC decreased from 56.23% to 47.63%. This was attributed to the intensification of the reactions of K_2_O and K_2_CO_3_ with the C atoms with increasing KOH addition [24,25]. However, the yields of the KBC_0.5:1_, KBC_1:1_, KBC_2:1_, and KBC_3:1_ samples did not substantially differ. The water absorption rates of the KBC_0.5:1_, KBC_1:1_, KBC_2:1_, and KBC_3:1_ samples were also analyzed (Table 1). The lowest water absorption rate (139.70%) was observed for KBC_2:1_.

With an increase in impregnation ratio to 3:1, the C content in the KBC increased from 80.51% to 90.23% and the O content decreased from 16.49% to 7.77% (Table 2). The decrease in the O content indicated that the occurrence frequency of the deoxygenation reaction increased with an increasing impregnation ratio [26]. At the same time, the contents of H and N decreased by 0.52% and 0.48%. The atomic ratio of H/C reflects the aromaticity of the biochar, while the atomic ratio of O/C reflects the hydrophilicity of the biochar [27]. With an increasing impregnation ratio, the H/C ratio decreased, indicating an increase in aromaticity. Concomitantly, the O/C ratio decreased, suggesting a decrease in hydrophilicity. This result was consistent with previous reports [28,29]. Based on the specific surface area, yield, water absorption rate, and elemental composition of the samples, the impregnation ratio of 2:1 was identified as the optimal condition.

### 2.2. Effect of Activation Temperature

**Figure 2:**
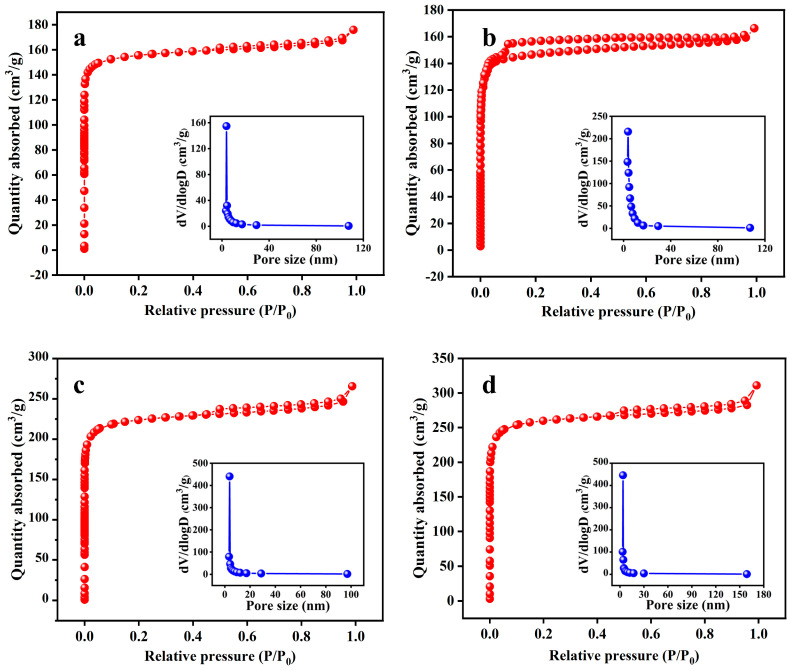
BET and BHJ pore−size distributions of KBC samples activated at various temperatures: (**a**) 600 °C, (**b**) 700 °C, (**c**) 800 °C, and (**d**) 900 °C.

The effects of various activation temperatures (600 °C, 700 °C, 800 °C, and 900 °C) on the properties (SSA, yield, water absorption rate, and elemental content) of KBC with an activation time of 2 h and impregnation ratio of 2:1 were investigated. The N_2_ adsorption–desorption isotherms of KBC_600_, KBC_700_, KBC_800_, and KBC_900_ (Figure 2) were type-I isotherms. KBC_700_ showed a significant H4 hysteresis loop for *P*/*P*_0_ = 0.3–1.0, while KBC_800_ and KBC_900_ exhibited significant H4 hysteresis loops at *P*/*P*_0_ = 0.4–1.0. When *P*/*P*_0_ was below 0.1, the adsorption capacity of KBC_600_, KBC_700_, KBC_800_, and KBC_900_ rapidly increased with increasing *P*/*P*_0_, indicating that all four samples contained numerous micropores. When *P*/*P*_0_ was between 0.1 and 0.9, the adsorption capacity of the four samples did not considerably change and the isotherms were almost horizontal, indicating the presence of macropores or mesopores. When *P*/*P*_0_ approached 1, a “tailing phenomenon” was observed for the four samples, confirming the presence of macropores or mesopores [30,31]. According to the BHJ pore-size distribution, the pore sizes of KBC_600_, KBC_700_, KBC_800_, and KBC_900_ were concentrated around 3.85 nm.

Because of the higher rate of the activation reaction and the release of more volatile substances at higher temperatures, the quality of the biochar gradually decreased with the increasing activation temperature. Therefore, with an increase in the activation temperature from 600 °C to 900 °C, the yield of KBC decreased from 62.03% to 39.53% [32]. In addition, the SSA of the KBC substantially increased with increasing temperature (Table 3). The KBC sample activated at 900 °C showed the highest SSA, micropore volume, total pore volume, and average pore size among the tested specimens; the corresponding values were 1032.361 m^2^/g, 0.310 cm^3^/g, 0.481 cm^3^/g, and 1.863 nm, respectively. This indicated that the pore structure of the KBC considerably improved with increasing activation temperature. According to the mechanism of activation by KOH, K_2_CO_3_ was generated during the process and reacted with the C in the material [33,34]. An increase in temperature was beneficial for this reaction because the melting points of K_2_CO_3_ and KOH are 891 °C and 380 °C, respectively. In addition, the Gibbs free energy of the reaction between KOH and C becomes negative at 570 °C, indicating that an increase in the activation temperature promoted the activation reaction [35]. KBC_900_ exhibited the lowest water absorption rate among the tested samples, which was 117.57% (Table 3).

Table 4 shows the contents of C, O, H, and N and their atomic ratios in the KBC samples activated at different temperatures. It can be seen that as the activation temperature increased from 600 °C to 900 °C, the C content in the materials considerably increased, while the O and H contents as well as the H/C and O/C atomic ratios substantially decreased. This was attributed mainly to the gradual decomposition of the organic matter (such as cellulose and hemicellulose) in the sunflower straws undergoing a series of reactions (including dehydration and cracking), accompanied by the volatilization of O and H [36]. With increasing activation temperature, the aromaticity of the KBC gradually increased and the hydrophilicity gradually decreased. Therefore, 900 °C was considered the optimal activation temperature.

### 2.3. Effects of Activation Time

The effects of different activation times (1, 2, and 3 h) on the properties (SSA, yield, water absorption rate, and elemental content) of KBC samples with an impregnation ratio of 2:1 and activated at 900 °C were investigated.

**Figure 3:**
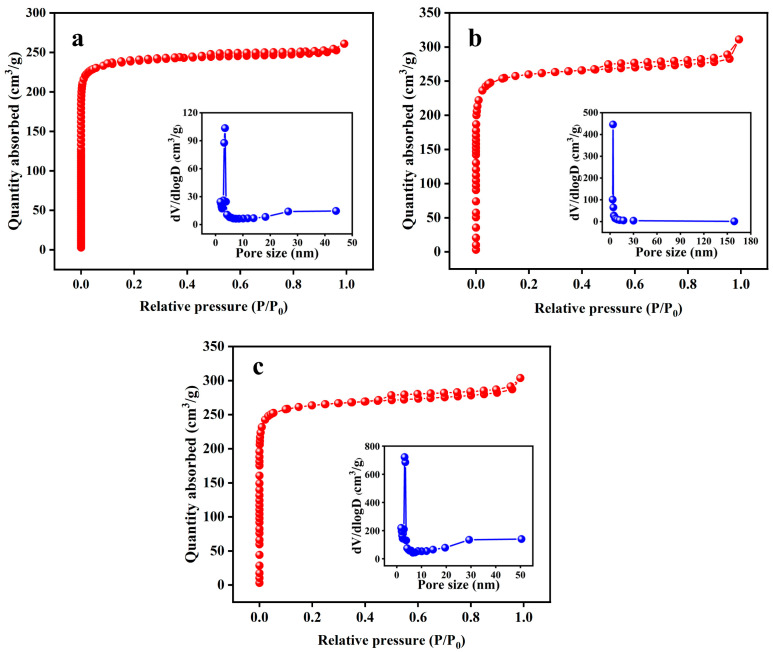
BET and BHJ pore−size distributions of KBC samples prepared for various activation times: (**a**) 1 h, (**b**) 2 h, and (**c**) 3 h.

The N_2_ adsorption–desorption isotherms of KBC_1_, KBC_2_, and KBC_3_ (Figure 3) were type-I isotherms. According to their BHJ pore-size distributions, the pore sizes of KBC_1_, KBC_2_, and KBC_3_ were concentrated between 3.48 and 3.82 nm.

As shown in Table 5, the SSAs of KBC_1_, KBC_2_, and KBC_3_ gradually increased and the micropore volumes did not considerably change with the extension of the activation time. Meanwhile, their average pore size and total pore volume first increased and then decreased with the extension of the activation time. The gradual increase in SSA indicated that the extension of the activation time promoted the formation of micropores. The formation of pores during activation was mainly divided into three steps: the expansion of existing pores, the formation of new pores, and the expansion of newly formed pores [37,38]. With the extension of the activation time, the reaction time between KOH and carbon inside the micropores increased, leading to the gradual expansion of existing micropores. Meanwhile, new micropores were generated. Therefore, the SSAs of the KBC increased with the extension of the activation time. However, the water absorption rate of the KBC first decreased and then increased with the extension of the activation time. The sample activated for 2 h showed the lowest water absorption rate, which was 117.57% (Table 5).

With the activation time extended to 2 h, the contents of C, O, and O/C as well as the H/C and H atomic ratios gradually decreased (Table 6), consistent with the results discussed in the previous section. However, with the activation time extended to 3 h, the contents of C, O, and H as well as the H/C and O/C atomic ratios did not considerably change. Therefore, based on the determined specific surface area, yield, and water absorption rate of the samples, 2 h was considered as the optimal activation time.

### 2.4. Characterization of KBC

#### 2.4.1. Micromorphology

**Figure 4:**
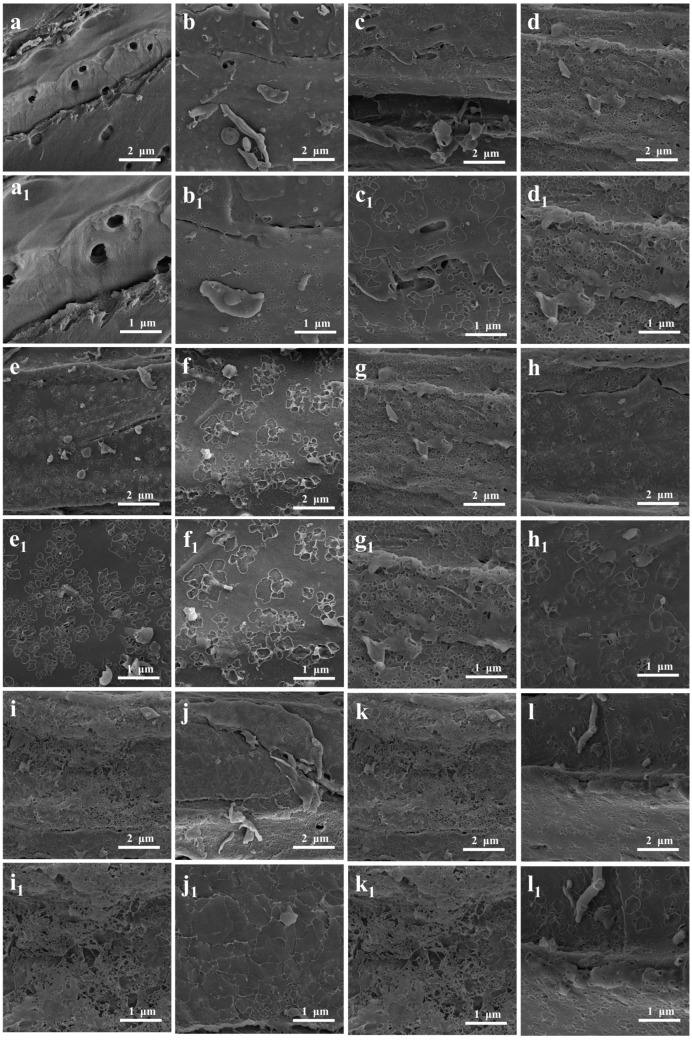
SEM images of (**a**,**a_1_**) SFSP, (**b**,**b_1_**) SFBC, (**c**,**c_1_**) KBC_1:1_, (**d**,**d_1_**) KBC_2:1_, (**e**,**e_1_**) KBC_3:1_, (**f**,**f_1_**) KBC_600_, (**g**,**g_1_**) KBC_700_, (**h**,**h_1_**) KBC_800_, (**i**,**i_1_**) KBC_900_, (**j**,**j_1_**) KBC_1_, (**k**,**k_1_**) KBC_2_, and (**l**,**l_1_**) KBC_3._

Figure 4a shows that the surface of the SFSP contained pits; nevertheless, the surface was overall smooth and had a low roughness. Meanwhile, the surface of the SFBC carbonized at 500 °C was rough and contained pores of different sizes (Figure 4b). After activation with KOH, even more irregular pore structures formed on the surface of the KBC, making the surface even rougher (Figure 4c–l). This result was attributed mainly to KOH, which damaged the fibers of the sunflower straws, and the uneven release of a large number of volatile substances, which damaged the surface of the SFBC [39]. Thus, activation with KOH increased the SSA and improved the pore structure of the SFBC (Figure 4(a_1_–l_1_)).

The KBC prepared with an impregnation ratio of 2:1 exhibited abundant pores and a rough surface (Figure 4d). It was consistent with the results obtained from SSA measurements mentioned earlier. With an increase in activation temperature, the pore structure on the surface of the KBC became more developed and surface roughness increased because of the decomposition of lignocellulose in the sunflower straws. The KBC activated at 900 °C showed a rougher surface than the KBC samples activated at other temperatures (Figure 4i), confirming the conclusions of the SSA analysis. In addition, the pore structure on the surface of the KBC activated for 1 h was rather undeveloped. The pore structure of the KBC activated for 2 h was more developed, with more microporous structures being formed. However, the KBC activated for 3 h exhibited a less developed pore structure than the KBC activated for 2 h (Figure 4l). This was because the metal oxides generated when the activation time was too long decomposed and blocked the already generated pores, destroying the pore structure.

#### 2.4.2. FTIR Results

**Figure 5:**
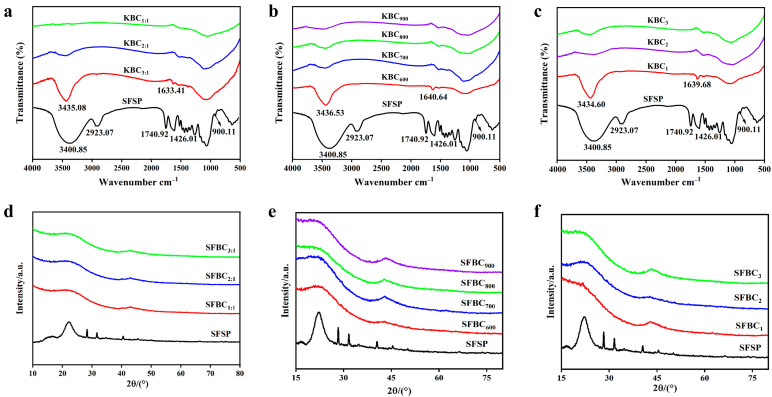
FTIR spectra and XRD patterns of KBC. (**a**–**c**) FTIR spectra of samples with different impregnation ratios, activation temperatures, and activation time, (**d**–**f**) XRD patterns of samples with different impregnation ratios, activation temperatures, and activation time.

The FTIR spectra of all the samples (Figure 5a–c) exhibited a peak related to the –OH stretching vibration at ~3400 cm^−1^. With the gradual increases in impregnation ratio, activation time, and temperature, the strength of this peak gradually decreased, indicating the increasing degree of biochar dehydration [40,41]. This also indicated that activation with KOH reduced the wettability of the biochar surface. With increasing degrees of activation, the intensity of the bands corresponding to alkane groups (–CH_3_ and –CH_2_) at ~2900 cm^−1^ gradually decreased. This was mainly attributed to the gradual decomposition of organic substances such as cellulose and hemicellulose in sunflower straws during activation, leading to the gradual removal of alkane groups. In addition, the presence of bands corresponding to C=O near the ~1740 cm^−1^ band indicated the presence of oxygen-containing functional groups (such as ester, carboxyl, and carbonyl groups) in sunflower straws [42]. The vibration band corresponding to C=O in the KBC shifted from around 1740 cm^−1^ to around 1640 cm^−1^. Its band intensity in the KBC was notably lower than that in the case of the sunflower straws, indicating a decreased amount of oxygen-containing functional groups in the biochar material [43]. This result was attributed to the easy breaking and conversion of C=O bonds near the ~1740 cm^−1^ wavenumber into CO and CO_2_ at high temperatures. Meanwhile, it also indicated the formation of aromatic structures on the surface of the KBC [44]. The spectral band near 900 cm^−1^ indicated the presence of aromatic structures. With an increase in the activation temperature from 600 °C to 900 °C, the number of C–C near ~1060 cm^−1^ and C–H near ~1420 cm^−1^ groups in the KBC samples gradually decreased. Meanwhile, with the extension of the activation time under a constant activation temperature, the degree of carbonization gradually increased and the number of C–C near ~1060 cm^−1^, C–H near ~1420 cm^−1^, and C=O near ~1640 cm^−1^ groups also decreased. The contents of –OH, C=O, and alkyl groups gradually decreased as the activation reaction proceeded, indicating a gradual increase in the aromatization degree of the KBC.

#### 2.4.3. XRD Results

The XRD patterns of KBC_1:1_, KBC_2:1_, KBC_3:1_, KBC_600_, KBC_700_, KBC_800_, KBC_900_, KBC_1_, KBC_2_, and KBC_3_ did not practically differ (Figure 5d–f). The diffraction peak around 2θ = 22° was wider for the KBC than for the SFSP. This peak was the characteristic diffraction peak of the (002) crystal plane of the carbon material, which indicated the presence of microcrystalline carbon in KBC. The presence of this microcrystalline structure endowed the KBC with a rich microporous structure, resulting in a large specific surface area [45]. This result was consistent with the specific surface area and SEM analysis results presented earlier. Additionally, the KBC exhibited a weak diffraction peak around 2θ = 43°, corresponding to the (220) crystal plane of KCI. This was because sunflower straws contain a high amount of K, which precipitated in the form of KCI at high activation temperatures. Notably, this characteristic diffraction peak was weaker and wider in KBC_900_ than in the other samples. This was attributed to the decrease in the crystallinity of KCI at high temperatures.

#### 2.4.4. XPS Results

**Figure 6:**
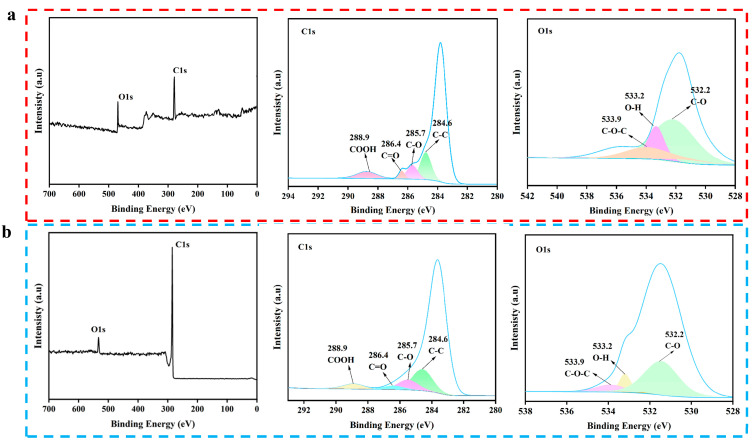
XPS spectra: (**a**) SFBC, (**b**) KBC.

To further explore the composition and changes in chemical functional groups, the SFBC and KBC were characterized by X-ray electron spectroscopy (XPS) (Figure 6). From the full spectra of the XPS, it could be seen that SFBC and KBC mainly contained two different elements: C and O. In order to further understand the composition of the SFBC and KBC, the functional groups of C and O were analyzed. The C1s spectra exhibited four main peaks: COOH (288.9 eV), C=O (286.4 eV), C–O (285.7 eV), and C–C (284.6 eV) [46]. The O1s spectra showed three main peaks: C–O–C (533.9 eV), O–H (533.2 eV), and C–O (532.2 eV). These results were consistent with the FTIR spectroscopy results. Compared with SFBC, the peaks corresponding to the C–O (285.7 eV), C=O (286.4 eV) and COOH (288.9 eV) bonds of the KBC were significantly weakened, while the peaks corresponding to C–C (284.6 eV) were wider. This indicated that the degree of graphitization was enhanced and the aromatic structure was enhanced. Moreover, the peak intensity corresponding to O–H (533.2 eV) was also weakened, indicating that the content of –OH was reduced after high-temperature activation. In general, high-temperature activation at 900 °C was beneficial to the graphitization degree of the biochar and reduced its content of oxygen-containing functional groups.

#### 2.4.5. Raman Analysis

**Figure 7:**
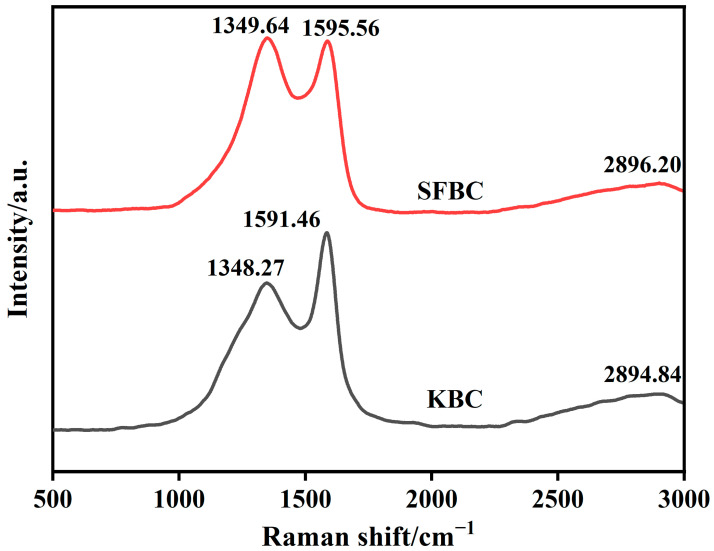
Raman spectra of SFSP, SFBC, and KBC.

Figure 7 shows the Raman spectra of the SFBC and KBC. The Raman spectra of the SFBC and KBC exhibited characteristic D and G peaks near 1350 and 1590 cm^−1^, respectively. The D peak represented condensed benzene rings in the biochar and the degree of defects in the C atomic structure. Meanwhile, the G peak represented sp^2^-hybridized C atoms in the biochar materials, reflecting the crystallinity and crystal symmetry of the biochar materials [47]. The I_D_/I_G_ ratio reflected the degree of graphitization of the biochar materials [48]. It could be seen that the I_D_/I_G_ ratio of SFBC was larger, indicating that the SFBC was mainly composed of amorphous carbon [49]. However, the D-peak intensity of the KBC sample decreased and the intensity of G peak increased and became sharp, which indicated that the ratio of I_D_/I_G_ decreased after high-temperature activation. It could be found from the figure that the ratio of I_D_/I_G_ was less than 1, indicating that the graphitization degree of the KBC increased. KBC exhibited a broad characteristic peak around 2890 cm^−1^, indicating the formation of graphitized structures during activation.

## 3. Material and Methods

### 3.1. Materials

Sunflower straws without moldy surfaces and with an average diameter of 2–3 cm were selected for the study. The samples were obtained from Bayannur, Inner Mongolia Autonomous Region, China. KOH was provided by Fuchen Chemical Reagent Co., Ltd. (Tianjin, China). Hydrochloric acid (HCl, 37 wt%) was procured from Yongfei Chemical Reagent Co., Ltd. (Langfang, Hebei, China). Distilled water was provided by Shanghai Lefeng Biotechnology Co., Ltd. (Hangzhou, China). All reagents were of analytically pure grade.

### 3.2. Instrumental Analysis

The sunflower straws were activated in a tube furnace (GSL-1500X, Hefei Kejing Material Technology Co., Ltd., China, Hefei, Anhui, China). The N_2_ adsorption isotherms of the samples were obtained using a fully automatic specific surface area tester (Micromeritics 3Flex, Micromeritics (Shanghai) Instrument Co., Ltd., Shanghai, China). The SSAs of the samples were calculated, and the pore-size distributions of the samples were determined using the Barrett–Joyner–Halenda (BJH) method. The surface morphology of the samples was observed using scanning electron microscopy (SEM; TESCAN MIRA LMS, Prague, Czech Republic). The absorption bands of the functional groups in the samples were determined using Fourier transform infrared (FTIR) spectroscopy in the potassium bromide compression mode (Nicolet iS20, Thermo Fisher Scientific, Waltham, MA, USA). The electronic binding energy of the samples was measured using X-ray photoelectron spectroscopy (XPS; Thermo Fisher Scientific-K-Al, Thermo Fisher Scientific, Waltham, MA, USA). The crystal structures of the samples were analyzed using X-ray diffraction (XRD; Smart Lab SE, Rigaku, Tokyo, Japan). The molecular vibrations, rotational energy, and symmetry of the samples were analyzed using Raman spectroscopy (Lab RAM HR Evolution, Horiba, Paris, France).

### 3.3. Preprocessing

Sunflower straws without mold were selected, and their pith was removed while retaining the xylem. The xylem was cut into sections with a length of approximately 10 cm and dried at 103 °C ± 2 °C in a drying oven to a constant weight. Sunflower straw powder (SFSP) with a particle size of 100 mesh was obtained by crushing the obtained dry straws in a multifunctional crusher and sieving through a 100-mesh screen.

### 3.4. Preparation of KBC

A certain amount of SFSP was weighed and placed in a crucible for pyrolysis in a tube furnace. The pyrolysis temperature was 500 °C, heating rate was 10 °C/min, and pyrolysis time was 2 h. Nitrogen was continuously supplied throughout the process. After pyrolysis, SFBC was removed from the furnace when the temperature dropped to 50 °C.

Subsequently, the KOH and SFBC were mixed at different impregnation ratios, distilled water was added, and the solution was continuously stirred for 4 h. After standing the solution for some time, the upper clear liquid was removed and the remaining solid was dried at 103 °C ± 2 °C for 5 h. The obtained samples were placed in a tube furnace and heat-treated at different temperatures for different periods. The set experimental conditions are summarized in Table 7. The heating rate in all the experiments was 10 °C/min. Throughout the activation process, nitrogen was continuously supplied into the furnace. After activation, the samples were removed from the furnace when the temperature dropped to 50 °C. The samples were then washed multiple times with distilled water and 0.1 mol/L HCl until a neutral pH was achieved and dried at 103 °C ± 2 °C for 8 h to obtain KBC. The KBC samples obtained under different activation conditions were referred to as (i) KBC_0.5:1_, KBC_1:1_, KBC_2:1_, and KBC_3:1_ (impregnation ratios of 0.5:1, 1:1, 2:1, and 3:1, respectively), the ratios represent the KOH–SFBC mass ratio; (ii) KBC_600_, KBC_700_, KBC_800_, and KBC_900_ (activation temperatures of 600 °C, 700 °C, 800 °C, and 900 °C, respectively); and (iii) KBC_1_, KBC_2_, and KBC_3_ (activation times of 1, 2, and 3 h, respectively).

### 3.5. Yield

A certain amount of SFBC was placed in a crucible, weighed (recorded as *M*_1_), activated, and weighed again (recorded as *M*_2_). The mass of the crucible was recorded as *M*. The yield (*Y*) was calculated according to Equation (1).

(1) $$Y\;\left(\%\right)=\frac{M_{2}-M}{M_{1}-M}\times 100\%$$

### 3.6. Water Absorption Rate

The water absorption rate was used to characterize the hydrophobicity of the samples. Specific tests were performed as follows. First, an empty conical flask was weighed (recorded as *M*_0_). Then, 2 g of the sample (*M*) was placed in a glass funnel with a filter paper and covered with a layer of gauze soaked in distilled water. Next, 20 g of distilled water was poured into the glass funnel, with the mass of the system recorded as *M*_1_. When the water droplets in the glass funnel stopped dripping, the mass of the conical flask and the distilled water in it was recorded as *M*_2_. Each sample was tested three times. The water absorption rate (*R*) was calculated according to Equation (2).

(2) $$R\;\left(\%\right)=\frac{{M_{1}-(M}_{2}-M_{0})}{M}\times 100\%$$

### 3.7. Specific Surface Area

An appropriate amount of the sample was weighed and degassed at 200 °C for 12 h. Subsequently, the N_2_ adsorption–desorption isotherm of the sample was obtained at 77 K using a fully automatic SSA tester. The SSA of the sample was determined via the BET method, and the pore-size distribution was determined using the BJH method.

## 4. Conclusions

Herein, we prepared biochar from sunflower straws and activated it using KOH. We determined the SSA, yield, water absorption rate, and elemental composition of the KBC prepared under various activation conditions, identifying the following ideal activation conditions: an activation time of 2 h, an activation temperature of 900 °C, and an impregnation ratio of 2:1. Under these conditions, the SSA of the biochar was 1032 m^2^/g, the yield was 39.53%, and the water absorption was 117.57%. The surface of the KBC became rougher, as analyzed by SEM. FTIR, XRD, XPS, and Raman characterization showed that its aromaticity and graphitization degree increased. The KBC prepared in this study has a high SSA and has good development potential in the adsorption of pollutants, energy utilization, and environmental protection.

## Figures and Tables

**Table 1:** Yield, water absorption rate, and pore structure of KBC samples with different impregnation ratios.

	*Y*/%	*R*/%	*A*_BET_ (m^2^/g)	*V*_mic_ (cm^3^/g)	*V* (cm^3^/g)	*D* (nm)
SFBC	0	337.83	4.034	0	0.026	25.806 ± 1.0
KBC_0.5:1_	56.23	198.10	506.812	0.139	0.227	2.109 ± 0.3
KBC_1:1_	50.40	165.27	404.555	0.191	0.258	2.239 ± 0.4
KBC_2:1_	48.43	139.70	594.655	0.213	0.267	1.676 ± 0.2
KBC_3:1_	47.63	156.13	284.456	0.0480	0.206	2.891 ± 0.5

Note: *Y*: yield; *R*: water absorption rate; *A*_BET_: BET surface area; *V*_mic_: micropore area; *V*: total pore volume; and D: average pore diameter.

**Table 2:** Elemental composition (wt%) and atomic ratios of KBC samples with different impregnation ratios.

	C (%)	O (%)	H (%)	N (%)	H/C	O/C
SFBC	63.39	30.57	2.50	3.54	0.47	0.36
KBC_1:1_	80.51	16.49	1.36	1.64	0.20	0.15
KBC_2:1_	85.28	12.14	1.10	1.48	0.15	0.11
KBC_3:1_	90.23	7.77	0.84	1.16	0.11	0.06

**Table 3:** Yield, water absorption rate, and pore structure of KBC samples activated at different temperatures.

	*Y*/%	*R*/%	*A*_BET_ (m^2^/g)	*V*_mic_ (cm^3^/g)	*V* (cm^3^/g)	*D* (nm)
SFBC	0	337.83	4.034	0	0.026	25.806 ± 1.0
KBC_600_	62.03	183.47	622.292	0.188	0.272	1.748 ± 0.1
KBC_700_	48.43	139.70	594.655	0.213	0.267	1.676 ± 0.2
KBC_800_	45.60	126.30	885.065	0.250	0.411	1.857 ± 0.4
KBC_900_	39.53	117.57	1032.361	0.310	0.481	1.863 ± 0.2

Note: *Y*: yield; *R*: water absorption rate; *A*_BET_: BET surface area; *V*_mic_: micropore area; *V*: total pore volume; and *D*: average pore diameter.

**Table 4:** Elemental contents (wt%) and atomic ratios in KBC samples activated at different temperatures.

	C (%)	O (%)	H (%)	N (%)	H/C	O/C
SFBC	63.39	30.57	2.50	3.54	0.47	0.36
KBC_600_	82.21	14.30	1.68	1.81	0.24	0.13
KBC_700_	85.28	12.14	1.10	1.48	0.15	0.11
KBC_800_	87.24	10.76	0.92	1.08	0.13	0.09
KBC_900_	92.48	5.62	0.77	1.13	0.10	0.05

**Table 5:** Yield, water absorption rate, and pore structure of KBC samples prepared for different activation times.

	*Y*/%	*R*/%	*A*_BET_ (m^2^/g)	*V*_mic_ (cm^3^/g)	*V* (cm^3^/g)	*D* (nm)
SFBC	0	337.83	4.034	0	0.026	25.806 ± 1.0
KBC_1_	44.4	154.87	961.719	0.312	0.404	1.640 ± 0.1
KBC_2_	39.53	117.57	1032.361	0.310	0.481	1.863 ± 0.2
KBC_3_	35.80	119.23	1052.600	0.331	0.470	1.787 ± 0.1

Note: *Y*: yield; *R*: water absorption rate; *A*_BET_: BET surface area; *V*_mic_: micropore area; *V*: total pore volume; and *D*: average pore diameter.

**Table 6:** Elemental contents (wt%) and atomic ratios in KBC samples prepared for different activation times.

	C (%)	O (%)	H (%)	N (%)	H/C	O/C
SFBC	63.39	30.57	2.50	3.54	0.47	0.36
KBC_1_	90.40	6.90	1.27	1.43	0.17	0.06
KBC_2_	92.68	5.62	0.77	0.93	0.10	0.05
KBC_3_	92.69	5.61	0.74	0.96	0.09	0.05

**Table 7:** Activation conditions of KBC samples.

	Impregnation Ratio	Activation Temperature (°C)	Activation Time (h)
1	0.5:1	600	1
2	1:1	700	2
3	2:1	800	3
4	3:1	900	—

## Data Availability

The data are contained within the article.
